# Time‐ and Temperature‐Dependent Response of Acquired FV Inhibitors: Implications for Laboratory Diagnosis and Clinical Management

**DOI:** 10.1002/jcla.70353

**Published:** 2026-09-15

**Authors:** Lihua Zhang, Mingjie Li, Zhiqiang Xie, Junxiang Yao, Yingping Cao, Lihui Chen, Meihua Wang

**Affiliations:** ^1^ Department of Laboratory Medicine, Fujian Medical University Union Hospital Fujian Medical University Fuzhou China; ^2^ School of Medical Technology and Engineering Fujian Medical University Fuzhou China; ^3^ Department of Laboratory Medicine Fujian Pingtan Comprehensive Experimental Area Hospital Fuzhou China

**Keywords:** blood coagulation factor inhibitors, blood coagulation tests, FV, mixing studies, temperature, time factors

## Abstract

**Background:**

Acquired factor V (FV) inhibitors are rare circulating autoantibodies that may cause severe bleeding and false‐negative detection without standardized incubation. This study aimed to evaluate time‐ and temperature‐dependent FV inhibitor activity and define optimal laboratory detection conditions.

**Methods:**

Fifteen serially diluted plasma samples from 4 FV inhibitor‐positive patients were incubated at room temperature (RT) or 37°C for 0–120 min. APTT mixing assays, residual FV activity, PT mixing assays, and Bethesda inhibitor titers were detected, with inhibitor‐free normal pooled plasma (NPP) as thermal degradation control. ISTH‐recommended MTC index was adopted for standardized mixing result interpretation.

**Results:**

At 37°C, APTT/PT mixing times were progressively prolonged and residual FV activity declined continuously, reaching a plateau at 60 min, whereas only slight changes occurred at RT. After excluding ~6% spontaneous FV thermal loss in NPP, residual FV activity at 37°C was significantly lower than RT at all time points (*p* < 0.05). Bethesda titers rose progressively at 37°C across all samples, confirming antibody‐mediated neutralization rather than native protein decay. Pronounced inter‑individual variability in inhibitor potency was observed under equalized inhibitor concentrations.

**Conclusions:**

FV inhibitors exhibit distinct time‐ and temperature‐dependent neutralizing activity. RT testing without sufficient 37°C incubation raises false‐negative risk. Universal 2 h incubation at 37°C is recommended for routine inhibitor screening; 60 min incubation is only suitable for follow‐up of confirmed FV inhibitor cases and cannot replace standard 2‐h protocol for ruling out FVIII inhibitors.

## Graphical abstract

This in vitro workflow illustrates the time‐and temperature‐dependent behavior of acquired FV inhibitors. Patient plasma was centrifuged and serially diluted, followed by parallel incubation at room temperature and 37°C. Serial measurements of APTT mixing test and residual FV activity were recorded across incubation time points to monitor inhibitor‐related changes under different experimental conditions.

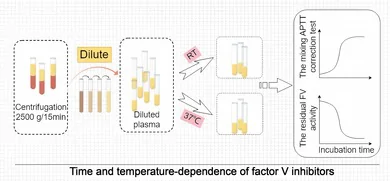

## 1 Introduction

Acquired inhibitors against coagulation factors represent a class of abnormal circulating antibodies that interfere with normal hemostasis. Among them, FVIII autoantibodies are the most prevalent, leading to acquired hemophilia A [1]. Acquired FV inhibitors represent an ultra‑rare autoimmune coagulation disorder that leads to acquired factor V deficiency, with an estimated prevalence of 0.09–0.29 cases per million individuals [1, 2]. This incidence rate is likely underestimated due to a substantial proportion of asymptomatic undiagnosed patients, and fewer than 200 relevant cases have been published globally, most as isolated case reports [2, 3]. Although the pathogenesis and clinical management of FVIII inhibitors have been thoroughly investigated [1, 4], the diagnostic properties and clinical implications of FV inhibitors remain poorly characterized, creating a prominent research gap.

FV is a single‐chain glycoprotein that acts as an essential cofactor of the prothrombinase complex and is vital for intact coagulation function [5, 6]. Autoantibodies directed against FV (termed FV inhibitors) directly bind to FV molecules and disrupt the clotting cascade, triggering a wide spectrum of bleeding phenotypes [3, 7, 8]. Clinical manifestations vary greatly among affected individuals, determined by residual FV activity, underlying precipitating diseases, and personal immune status. Mild presentations include subcutaneous ecchymosis and mucosal hemorrhage (gingival, nasal bleeding), while life‐threatening visceral or intracranial hemorrhage may occur in severe cases [7, 8]. Notably, thrombotic events have also been reported alongside bleeding diatheses, which complicates clinical diagnosis and therapeutic decision‐making [9, 10].

Numerous studies have validated the time‐ and temperature‐dependent neutralizing activity of FVIII inhibitors; incubation at 37°C for 2 h is now the gold standard for routine FVIII inhibitor detection. Nevertheless, studies focusing on other coagulation factor inhibitors are scarce. The mainstream consensus holds that non‐FVIII inhibitors bind and neutralize their target factors instantaneously without relying on incubation time or temperature. Isolated case reports have implied potential temperature sensitivity of FV inhibitors, yet no systematic gradient incubation and serial dilution experiments have been performed to verify this hypothesis. To fill this unmet research need, we designed a standardized in vitro experiment to systematically characterize the time‐ and temperature‐dependent kinetic behaviors of FV inhibitors across multiple titer ranges, and to establish optimized laboratory protocols to boost diagnostic accuracy for this rare disorder.

## 2 Materials and Methods

### 2.1 Samples Collection

Four patients diagnosed with acquired coagulation FV deficiency were included in this study. Patients 1, 2, and 4 were admitted due to abnormal coagulation test results, whereas Patient 3 was enrolled due to persistent coagulopathy following colorectal cancer surgery. Plasma samples were collected from these 4 confirmed FV inhibitor‐positive patients at Fujian Medical University Union Hospital between January 2022 and May 2024.

Peripheral blood samples were collected into tubes containing 3.2% sodium citrate anticoagulant (9:1 blood‐to‐anticoagulant ratio). Samples were centrifuged twice at 2500 × g for 15 min to obtain platelet‐poor plasma (PPP), which was immediately utilized for subsequent assays or stored at −80°C if testing was delayed. Both undiluted and serially diluted plasma samples (diluted with STA‑Deficient V reagent (FV‑deficient plasma)) were tested on the day of blood collection to evaluate the APTT mixing assay, residual FV activity, and PT mixing assay. A total of 15 serially diluted plasma samples derived from 4 patients with acquired FV inhibitors with various inhibitor titers were analyzed; imidazole buffer solution was used for Bethesda inhibitor quantification. Full raw laboratory data are detailed in Supplementary Table S1.

### 2.2 Determination of FV Inhibitors

FV inhibitors were quantified strictly according to the Bethesda assay method previously described [4], involving incubation of plasma samples at 37°C for 2 h to ensure accurate detection of inhibitory antibodies. Briefly, pooled normal human plasma was prepared and mixed with an equal volume of imidazole buffer solution as a reference control. Patient plasma samples were serially diluted with imidazole buffer at dilution ratios of 1:1, 1:2, 1:4, etc. Each diluted patient plasma sample was mixed with an equal volume of pooled normal human plasma and incubated at 37°C for 2 h. Residual FV activity was then measured, and inhibitor titers were calculated. One Bethesda Unit (BU) is defined as the amount of inhibitor that reduces FV activity by 50% under standardized conditions. In addition to the standard 2 h incubation, we quantified FV inhibitor titers at serial incubation time points (0, 15, 60, 120 min) under both RT and 37°C to directly characterize time‐ and temperature‐dependent antibody activity.

### 2.3 APTT Mixing Assay

APTT mixing assay was performed using an automated coagulation analyzer (STA‐R Evolution; Diagnostica Stago, Asnières‐sur‐Seine, France) employing mechanical clot detection, unaffected by lipemia, hemolysis, or icterus. Normal pooled plasma was prepared from equal volumes of plasma samples obtained from 20 healthy donors, explicitly excluding individuals with recent infection, malignancy, trauma, surgery, or pregnancy, and confirmed to have normal coagulation profiles.

Patient plasma samples were mixed with normal pooled plasma at a 1:1 ratio. The mixtures were incubated at either room temperature (RT) or 37°C for designated intervals of 0, 15, 30, 60, and 120 min. After incubation, APTT mixing times were measured using STA‐PTT A reagent (REF 00595; Diagnostica Stago).

Interpretation of APTT mixing results followed the Chinese Guidelines for Diagnosis and Treatment of Coagulation FVIII/IX Inhibitors (2018 Edition) and the Chinese Expert Consensus on the Operational Procedures and Result Interpretation of the Prolonged Mixed Plasma Assay for Activated Partial Thromboplastin Time. An APTT mixing time ≥ 42.0 s (normal reference range: 28.0–42.0 s) indicated incomplete neutralization, consistent with circulating coagulation inhibitors. Original result interpretation relied on institutional qualitative cut‐offs. Following ISTH recommendations for mixing assay evaluation, we further calculated the quantitative mixing assay‐specific cut‐off (MTC) index according to Devreese et al. [4] to better distinguish factor deficiency and inhibitory antibodies and improve the generalizability of our laboratory data. MTC = 1:1 mixture APTT mixing time/normal pooled plasma APTT time, with MTC > 1.07 defined as positive for incomplete neutralization. All incubation groups were assessed using this quantitative index.

### 2.4 Determination of Residual FV Activity

FV activity was determined using a one‐stage clotting assay based on PT, performed on an automated coagulation analyzer (STA‐R Evolution; Diagnostica Stago). The assay utilized STA‐Deficient V reagent (REF 00744; Diagnostica Stago), which specifically contains FV‐deficient plasma with all other coagulation factors present in excess. PT measurements were performed using STA‐Neoplastine CI Plus reagent (REF 00606, REF 00667; Diagnostica Stago), with additional control reagents STA‐Owren‐Koller buffer (REF 00360) and STA‐System Control (REF 00678).

A calibration curve (FV:C_PT) was generated using STA‐Unicalibrator reagent (REF 00675; Diagnostica Stago), traceable to international reference standards. Patient plasma samples were analyzed at multiple dilutions to confirm parallelism and avoid interference from high inhibitor titers, ensuring accurate FV activity measurement. Consequently, the measured PT directly reflected FV activity levels in the tested plasma. FV activity was expressed as a percentage based on this calibration curve.

### 2.5 PT Mixing Assay

PT was determined separately using the STA‐Neoplastine CI Plus reagent (REF 00606, REF 00667; Diagnostica Stago) according to the manufacturer's instructions. Patient plasma samples were mixed with normal pooled plasma at a 1:1 ratio similar to the mixing APTT assay. The mixtures were incubated at room temperature (RT) and 37°C for intervals of 0, 15, 30, 60, and 120 min following previously described incubation protocols. PT values reflected time‐ and temperature‐dependent FV inhibitor effects.

### 2.6 Normal Pooled Plasma Thermal Degradation Control Experiment

To distinguish spontaneous thermal degradation of intrinsic FV protein from antibody‐mediated FV neutralization by FV inhibitors, inhibitor‐free normal pooled plasma (NPP, derived from the same 20 healthy donors as the mixing assay matrix) was selected as the negative control. To eliminate bias caused by excessively high FV activity in undiluted neat NPP, all normal plasma aliquots were serially diluted with STA‑Deficient V reagent (FV‑deficient plasma) following the identical dilution gradients used for patient plasma samples prior to incubation. Both diluted NPP and serially diluted patient specimens underwent parallel incubation at room temperature (RT) and 37°C for 0, 15, 30, 60, and 120 min. After each incubation interval, APTT mixing time, residual FV activity, and PT mixing time were determined using consistent assay protocols described above. The degree of spontaneous FV activity decline in diluted NPP was calculated to establish a baseline threshold of native FV thermal lability, which was used to differentiate protein autodegradation from antibody‐driven FV inactivation in patient samples. All thermal degradation data for diluted inhibitor‐free NPP are summarized in Table S2, and full datasets of serially diluted patient plasma are provided in Table S1.

### 2.7 Statistical Analysis

Statistical analyses were performed using SPSS version 22.0 (SPSS Inc., Chicago, IL, USA). GraphPad Prism 9.0 software (GraphPad Software Inc., San Diego, CA, USA) was used to generate plots illustrating the time‐ and temperature‐dependent trends of FV inhibitor activity. The paired *t*‐test or Wilcoxon matched‐pairs signed‐rank test was applied to compare results from the APTT mixing assays and residual FV activity assays across different incubation durations and temperatures, depending on the normality and variance homogeneity of the data. Statistical significance was defined as a *p*‐value < 0.05.

## 3 Results

### 3.1 Clinical Characteristics

This study enrolled 4 patients with laboratory‐confirmed acquired FV inhibitors collected between January 2022 and May 2024 at Fujian Medical University Union Hospital. All participants presented isolated prolongation of PT and APTT, whereas thrombin time (TT) stayed within the normal reference range. Lupus anticoagulant and antiphospholipid antibody screening were negative for all patients, ruling out antiphospholipid syndrome as the cause of coagulopathy. Routine coagulation factor testing showed markedly reduced FV activity in each subject. Infusion of fresh frozen plasma (FFP) and cryoprecipitate failed to correct their coagulation abnormalities, further indicating the existence of circulating neutralizing inhibitors.

Individual clinical backgrounds and inhibitor titers are summarized as follows:

Patient 1: Acquired FV inhibitor likely triggered by autoimmune hepatitis, baseline inhibitor titer   = 17.6 BU/mL.

Patient 2: Multiple predisposing factors including β‐lactam antibiotic exposure, baseline inhibitor titer = 90 BU/mL.

Patient 3: Acquired FV inhibitor secondary to colorectal cancer surgery, baseline inhibitor titer = 1.6 BU/mL.

Patient 4: History of β‐lactam administration before coagulation disorder onset,baseline inhibitor titer = 274 BU/mL.

Standard Bethesda assays combined with 37°C 2‐h incubated mixing tests confirmed time‐dependent inhibitor patterns in all 4 subjects, with > 10 s difference in APTT clotting time between immediate and delayed mixing tests, consistent with time‐ and temperature‐sensitive autoantibodies.

A total of 15 serially diluted plasma samples derived from these 4 patients were subjected to gradient incubation experiments. Full raw data covering APTT mixing time, residual FV activity, and PT values at all time–temperature points are listed in Table S1. Parallel thermal degradation data of diluted inhibitor‐free normal pooled plasma (NPP) for baseline correction are summarized in Table S2.

### 3.2 Effects of Time and Temperature on the APTT Mixing Assay

Results from the APTT mixing assay showed a clear trend of progressively prolonged mixing times with increasing incubation durations, observed at both room temperature (RT) and 37°C, until a plateau was eventually reached (Figure 1A,B). In terms of temperature‐dependent effects, APTT mixing times were significantly prolonged at 37°C compared to room temperature (Figure 1C). When examining time‐dependent trends, a stepwise increase in APTT mixing time was observed with longer incubation durations at 37°C. However, this increase did not reach statistical significance between 60 and 120 min at 37°C (both *p* = 0.05; Figure 1D). Notably, parallel incubation of inhibitor‐free normal pooled plasma excluded spontaneous FV thermal decay as a major contributor to this trend, as NPP displayed negligible APTT prolongation throughout the entire incubation period.

**FIGURE 1:**
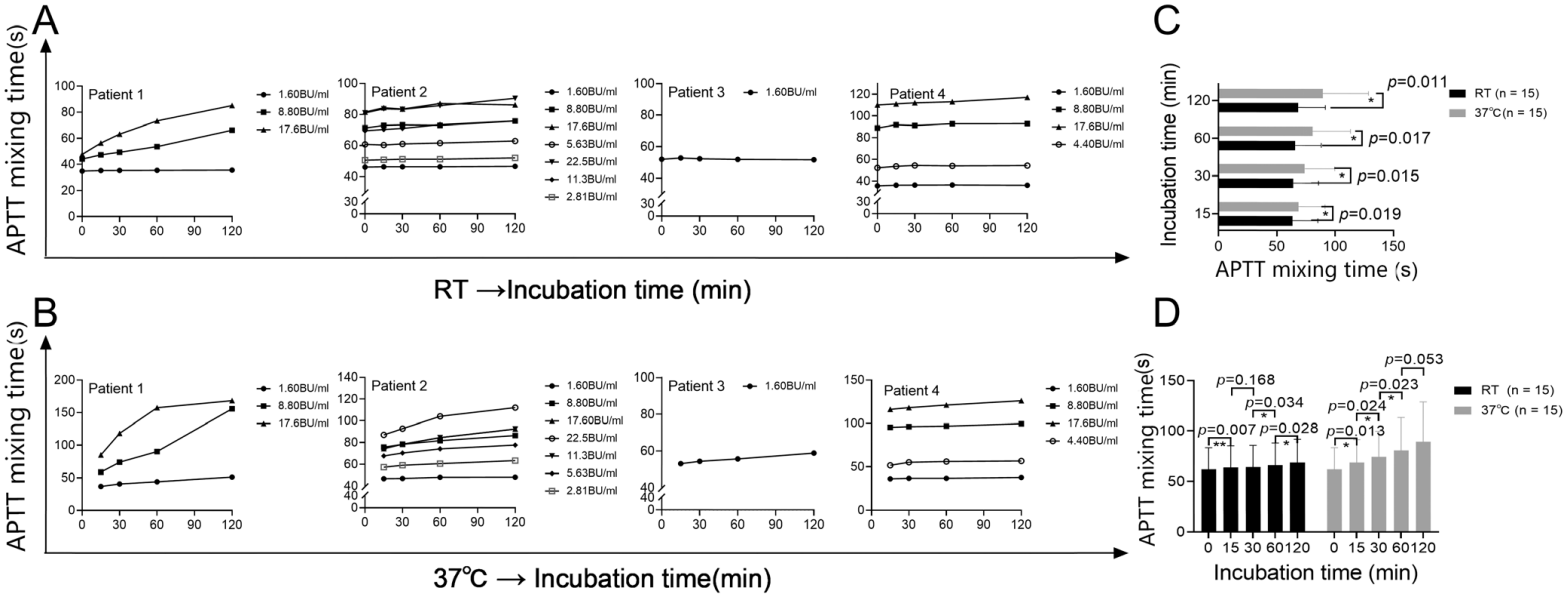
Time‐dependent changes in APTT mixing time at room temperature and 37°C APTT mixing assay performed at RT (A) and 37°C (B) with varying incubation time intervals. (C) Statistical comparison of APTT mixing results between RT and 37°C. (D) Statistical comparison of APTT mixing results across different incubation time intervals. *p* < 0.05 was defined as statistically significant. *n* = 15 serially diluted samples derived from 4 patients with acquired FV inhibitors.

### 3.3 Effects of Time and Temperature on Residual FV Activity

An inverse relationship between residual FV activity and APTT mixing times was clearly observed (Figure 2A,B). Residual FV activity declined significantly with longer incubation durations at both room temperature (RT) and 37°C, ultimately reaching a plateau (Figure 2D). Additionally, FV activity levels showed a negative correlation with inhibitor titers. Interestingly, even when FV inhibitor concentrations were equalized through dilution, the degree of FV activity reduction varied substantially between patient samples, highlighting marked inter‐individual differences in inhibitor behavior.

**FIGURE 2:**
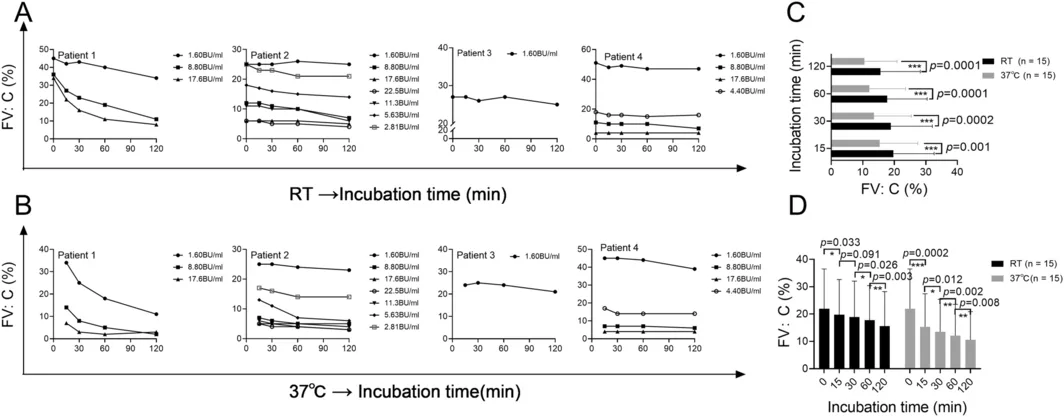
Time‐dependent changes in residual FV activity at room temperature and 37°C Residual FV activity measured at RT (A) and 37°C (B) across serial incubation durations. (C) Statistical comparison of residual FV activity between RT and 37°C. (D) Statistical comparison of residual FV activity at different incubation time points. *p* < 0.05 was defined as statistically significant. *n* = 15 serially diluted samples derived from 4 patients with acquired FV inhibitors.

When comparing equivalent FV inhibitor titers at different incubation temperatures, residual FV activity consistently declined more markedly at 37°C than at RT (*p* < 0.05; Figure 2C), with Patient 1 exhibiting the most substantial reduction (Figure 2A,B, Table S1). The magnitude of residual FV activity reduction in all patient specimens far exceeded the ~6% spontaneous activity loss detected in inhibitor‐free normal pooled plasma following 120 min incubation at 37°C, verifying antibody‐mediated neutralization rather than intrinsic protein instability as the primary driver of these changes. Pooled analysis of all patient‐derived diluted samples demonstrated that residual FV activity levels were significantly lower at 37°C than at room temperature across all tested incubation intervals after excluding the ~6% spontaneous FV activity loss observed in inhibitor‐free normal pooled plasma.

### 3.4 Time‐ and Temperature‐Dependent Changes of FV Inhibitor Bethesda Titers

Consistent with the dynamic reduction of residual FV activity, Bethesda unit (BU) values of all 15 diluted samples exhibited obvious time‐ and temperature‐dependent rising trends under parallel incubation conditions. At 37°C, FV inhibitor titers increased continuously from 0 min to 120 min; in contrast, only mild, marginal elevations of inhibitor levels were detected at room temperature at the same time intervals. Samples with intermediate baseline inhibitor concentrations (8.8–11.3 BU/mL) presented the most prominent upward shifts in BU values during incubation. For samples with extremely low initial titers (1.6 BU/mL), insufficient circulating autoantibodies limited further inhibitor accumulation, while samples with high baseline titers (> 17.6 BU/mL) nearly exhausted free FV at time zero, resulting in blunted titer elevation after prolonged incubation. These parallel upward changes in inhibitor BU values, together with the marked drop of residual FV activity after incubation, provide direct quantitative evidence that prolonged incubation at 37°C strengthens the neutralizing capacity of FV autoantibodies, rather than only inducing spontaneous thermal decay of native FV. Notably, the inhibitor‐free normal pooled plasma used as negative control underwent identical serial dilution matching patient plasma gradients to balance basal FV levels, avoiding confounding from high native FV concentration in undiluted control material.

### 3.5 Effects of Time and Temperature on the PT Mixing Assay

PT measurements also demonstrated a clear time‐ and temperature‐dependent pattern, particularly under incubation at 37°C. Among the 15 analyzed samples, PT values increased progressively from 0 to 60 min and showed a modest but consistent rise through 120 min. For instance, in Patient 1 (17.6 BU/mL), PT extended from 17.9 s at 60 min to 22.8 s at 120 min; similarly, in Patient 2 (17.6 BU/mL), values increased from 31.9 to 34.9 s over the same interval (Figure 3A,B). Paired statistical comparisons between the 60‐ and 120‐min time points at 37°C revealed significant differences, as confirmed by both the paired *t*‐test (*p* = 0.00043) and the Wilcoxon signed‐rank test (*p* = 6.1 × 10^−5^), indicating continued, albeit tapering, FV inactivation over time (Figure 3D). At each matched incubation time point, PT mixing times were significantly longer at 37 °C compared with room temperature (Figure 3C).

**FIGURE 3:**
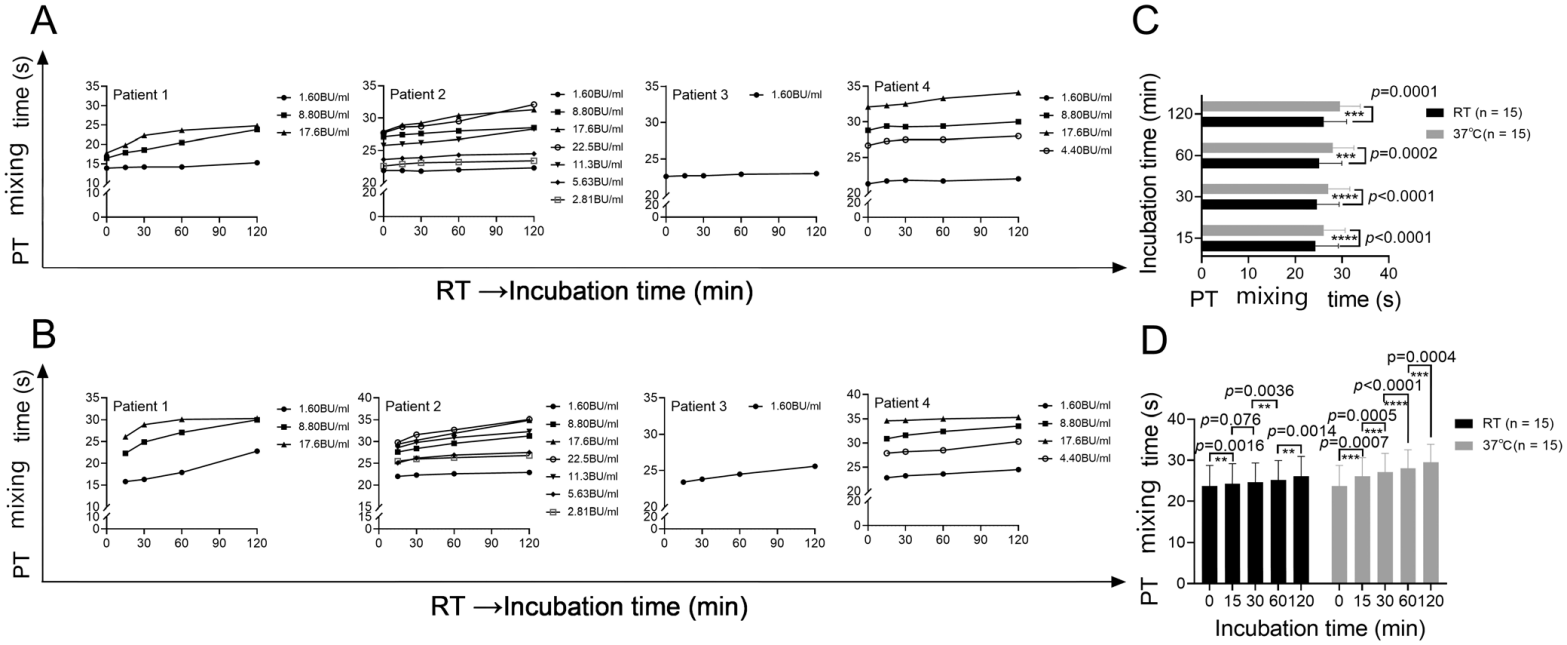
Time‐dependent changes in PT mixing time at room temperature and 37°C PT mixing assay performed at RT (A) and 37°C (B) with varying incubation time intervals. (C) Statistical comparison of PT mixing results between RT and 37°C. (D) Statistical comparison of PT mixing results across different incubation time intervals. *p* < 0.05 was defined as statistically significant. *n* = 15 serially diluted samples derived from 4 patients with acquired FV inhibitors.

### 3.6 Normal Pooled Plasma Thermal Degradation Control Experiment

To eliminate the confounding effect of spontaneous thermal degradation of native FV protein, inhibitor‐free normal pooled plasma (NPP) from 20 healthy donors was incubated in parallel with all patient plasma under identical room temperature (RT) and 37°C incubation protocols (0, 15, 30, 60, 120 min). Only mild spontaneous loss of FV activity was observed in NPP after 120 min incubation at 37°C, with a maximum activity reduction of approximately 6% relative to baseline at 0 min. No time‐dependent prolongation of APTT mixing time or PT mixing time was identified in NPP across all incubation intervals.

Intergroup statistical comparisons confirmed that the reduction rates of residual FV activity in all patient plasma samples were significantly higher than those of NPP at every incubation time point at 37°C (all *p* < 0.001). For patient plasma with intermediate inhibitor titers (8.8–11.3 BU/mL), FV activity declined by 40%–88% after 120 min incubation at 37°C, far exceeding the minor spontaneous degradation of NPP. Samples with extremely low (1.6 BU/mL) or high (> 17.6 BU/mL) inhibitor titers showed blunted kinetic responses, yet their FV activity loss still exceeded the 6% degradation threshold of normal plasma controls. These findings confirm that the progressive FV inactivation and clotting time prolongation observed in patient specimens were predominantly driven by time‐ and temperature‐dependent neutralization mediated by FV autoantibodies, rather than intrinsic thermal lability of FV protein. All thermal degradation raw data for NPP are listed in Table S2, while full serially diluted patient plasma datasets are available in Table S1.

## 4 Discussion

The molecular pathogenesis of acquired coagulation FV deficiency remains incompletely understood. Historically, many reported cases of acquired FV inhibitors were associated with surgical exposure to bovine‐derived topical hemostatic thrombin preparations [7, 8, 11]. With the gradual elimination of bovine thrombin in clinical practice, the incidence of this iatrogenic complication has declined, yet FV autoantibodies still represent a critical diagnostic challenge in contemporary laboratories. Multiple predisposing triggers have been documented in recent literature, including broad‐spectrum antibiotics (predominantly β‐lactams), malignant tumors, surgical trauma, autoimmune disorders, infections, and organ transplantation; roughly 30% of patients present with idiopathic FV inhibition without identifiable precipitating factors [12, 13, 14]. In this cohort, three of 4 patients received β‐lactam antibiotics prior to coagulation dysfunction, and one suffered postoperative colorectal malignancy‐related bleeding, consistent with previously reported risk associations. Antibiotics may induce conformational alterations in FV epitopes to trigger de novo autoantibody production [10]. The clinical spectrum of FV inhibitor patients is highly heterogeneous, ranging from asymptomatic laboratory abnormalities to life‐threatening intracranial or visceral hemorrhage, while concurrent thrombotic events are also occasionally observed, further complicating clinical judgment [9, 10]. Reported overall mortality ranges from 14% to 31%, underscoring the necessity of timely, accurate laboratory identification [15, 16].

Acquired FVIII inhibitors have been thoroughly characterized to possess prominent time‐ and temperature‐dependent neutralizing capacity; current gold‐standard protocols mandate 2 h incubation at 37°C to maximize inhibitor detection sensitivity [17, 18]. In contrast, most prior literature presumed that non‐FVIII coagulation inhibitors exert immediate target protein neutralization independent of incubation conditions, with minimal systematic experimental validation for FV autoantibodies [5, 9, 19]. Our serial gradient incubation data challenge this long‐standing assumption. Across all 15 serially diluted plasma specimens from 4 inhibitor‐positive patients, prolonged incubation at physiological temperature progressively elevated Bethesda inhibitor titers, reduced residual FV activity, and prolonged PT/APTT mixing times, whereas only trivial changes occurred at room temperature. This consistent kinetic trend across all titer subgroups confirms that acquired FV inhibitors exhibit analogous temperature‐ and time‐sensitive behavior to FVIII autoantibodies.

A core methodological concern raised by peer reviewers is whether the observed FV activity loss merely reflects intrinsic thermal lability of native FV rather than antibody‐mediated neutralization. We rigorously controlled for this confounding factor by implementing matched, serially diluted inhibitor‐free normal pooled plasma (NPP) as parallel blanks, with dilution gradients identical to patient samples to eliminate bias from undiluted high baseline FV concentrations. After 120 min incubation at 37°C, NPP only exhibited approximately 6% spontaneous FV degradation without meaningful clotting time prolongation. In stark contrast, intermediate‐titer patient samples (8.8–11.3 BU/mL) demonstrated 40%–88% FV inactivation after identical incubation, with all patient subgroups showing significantly greater FV loss than the NPP baseline threshold (all *p* < 0.001). The concurrent continuous rise in Bethesda titers over incubation provides direct quantitative proof that FV autoantibody neutralization, not spontaneous protein decay, drives the observed coagulation abnormalities.

The variable magnitude of time‐dependent responses across patients stems from inhibitor titer kinetics rather than differential FV thermal breakdown. Intermediate inhibitor concentrations yielded the most robust temperature‐activated neutralization. Low‐titer specimens (1.6 BU/mL) lacked sufficient circulating autoantibodies to generate pronounced kinetic shifts, while high‐titer samples (> 17.6 BU/mL) nearly exhausted available FV at time zero, leaving minimal room for further antibody‐mediated suppression. Even patients with milder overall responses displayed FV activity declines exceeding the 6% spontaneous degradation cut‐off, verifying genuine temperature‐dependent autoantibody function. Inter‐individual disparities in inhibitory sensitivity likely arise from differences in antibody avidity and targeted FV epitopes, as documented in prior mechanistic studies [6, 20, 21, 22]. A previously published case report similarly described misdiagnosis of congenital FV deficiency when mixing assays were performed without extended 37°C incubation; only delayed incubation successfully identified the underlying acquired FV inhibitor [3], aligning with our experimental conclusions.

High‐titer FV inhibitors cause artifactual reductions in multiple coagulation factor activities on one‐stage clotting assays, as prolonged PT/APTT impairs the readout of all common pathway factors (Table S1). This analytical interference must be distinguished from true combined factor deficiencies through standardized incubated mixing testing. In this work, we abandoned institutional qualitative mixing cut‐offs and adopted the quantitative mixing test‐specific cut‐off (MTC) index recommended by ISTH guidelines [4]. This standardized numerical metric eliminates subjective inter‐laboratory interpretative bias and improves cross‐study comparability, enabling clearer differentiation between inherited factor deficiency and circulating inhibitory autoantibodies.

Clinically critical guidance must be stratified for distinct laboratory testing scenarios. Although obvious FV inhibitory kinetics emerge after 60 min incubation at 37°C for patients with a confirmed diagnosis of acquired FV deficiency, this shortened incubation protocol cannot be adopted for universal unknown coagulation screening. Time‐dependent FVIII inhibitors require full 2 h co‐incubation to reach maximal neutralizing activity; laboratories encountering unclassified prolonged clotting results must uniformly apply the 37°C/2 h standard incubation to avoid missed acquired hemophilia A diagnoses. The 60 min shortened incubation is only appropriate for follow‐up monitoring of already confirmed FV inhibitor cases and cannot replace the universal 2 h screening workflow.

This study possesses several inherent limitations that require transparent acknowledgment. First, the cohort comprises only 4 FV inhibitor patients, which restricts the external generalizability of our laboratory recommendations; large‐scale, multicenter prospective cohorts are required to further validate the titer‐dependent kinetic patterns identified herein. Second, all observations originate from static in vitro incubation assays, without longitudinal in vivo monitoring of inhibitor levels following immunosuppressive therapy. Third, we did not conduct antibody epitope and avidity testing, which prevents full elucidation of the molecular basis for inter‐patient variability in inhibitory responsiveness.

Our systematic serial dilution and gradient incubation in vitro experiments definitively demonstrate robust time‐ and temperature‐dependent neutralizing activity of acquired FV autoantibodies, which cannot be attributed solely to spontaneous thermal degradation of native FV protein. Performing mixing assays and Bethesda testing exclusively at room temperature without sufficient 37°C pre‐incubation substantially elevates false‐negative risk and risks misdiagnosis of congenital FV deficiency. We recommend universal adoption of 37°C for 2 h incubation for all routine coagulation inhibitor screening. Combined application of serial plasma dilution, ISTH‐standardized MTC quantitative index, and matched diluted normal plasma blank controls effectively separates autoantibody‐mediated inhibition from intrinsic factor lability or true factor deficiency, facilitating timely diagnosis and targeted immunosuppressive intervention for patients with acquired FV deficiency.

## Author Contributions

Lihua Zhang: performed the experiments, analyzed the data, prepared the figures, and drafted the manuscript. Mingjie Li: collected patient samples and assisted with data analysis. Zhiqiang Xie and Junxiang Yao: conducted laboratory testing and assisted with data collection. Yingping Cao: participated in data sorting and preliminary statistical analysis. Lihui Chen: provided experimental supervision, interpreted laboratory results, and revised the manuscript critically. Meihua Wang: conceived and designed the overall research project, obtained research funding, and finalized the approved manuscript for submission.

## Funding

No funding was received for this study.

## Ethics Statement

This study was approved by the Ethics Committee of Fujian Medical University Union Hospital (Approval number: 2022003) and conducted in strict accordance with the Declaration of Helsinki.

## Consent

Written informed consent was obtained from all participants.

## Conflicts of Interest

The authors declare no conflicts of interest.

## Supporting information


**Table S1:** PT‐mixing time, APTT‐mixing time and residual FV activity of 15 diluted plasma samples derived from 4 patients with acquired factor V inhibitors under room‐temperature (RT) and 37°C incubation at different time points.
**Table S2:** Spontaneous thermal degradation data of inhibitor‐free pooled control plasma incubated at room temperature and 37°C for 0–120 min.

## Data Availability

The data that support the findings of this study are available on request from the corresponding author. The data are not publicly available due to privacy or ethical restrictions.
