# Supplementary material for: Time‐ and Temperature‐Dependent Response of Acquired FV Inhibitors: Implications for Laboratory Diagnosis and Clinical Management
Source: J Clin Lab Anal. 2026 Sep 15;40(20):e70353. doi: 10.1002/jcla.70353 (PMC13576664; doi:10.1002/jcla.70353)
Supplement: Supplementary file 1 [file JCLA-40-e70353-s001.docx]

| **Diluted samples (n= 15)** | **Incubation RT (min)** | | | | | **Incubation 37℃ (min)** | | | |
| --- | --- | --- | --- | --- | --- | --- | --- | --- | --- |
|  | **0** | **15** | **30** | **60** | **120** | **15** | **30** | **60** | **120** |
|  | APTT mixing time (s) | | | | | | | | |
| Patient1(1.6BU/mL) | 34.8 | 35.2 | 35.3 | 35.4 | 35.5 | 36.9 | 40.7 | 44.1 | 51.4 |
| Patient 1(8.8 BU/mL) | 44.0 | 47.3 | 49.3 | 53.6 | 66.2 | 58.8 | 73.9 | 90.2 | 156 |
| Patient1(17.6 BU/mL) | 47.6 | 56.4 | 63.2 | 73.3 | 85.0 | 85.1 | 118 | 157 | 168 |
| Patient2(1.6BU/mL) | 46.0 | 46.2 | 46.2 | 46.2 | 46.5 | 46.8 | 47.0 | 48.1 | 48.2 |
| Patient 2(8.8 BU/mL) | 71.3 | 72.9 | 73.3 | 72.9 | 75.7 | 75.7 | 78.3 | 81.7 | 86.4 |
| Patient 2(17.6 BU/mL) | 81.5 | 84.3 | 83.4 | 87.0 | 86.1 | 86.1 | 91.2 | 99.1 | 107 |
| Patient 2(22.5 BU/mL) | 80.9 | 83.6 | 83.2 | 85.7 | 90.4 | 87.0 | 92.7 | 104 | 112 |
| Patient 2(11.3 BU/mL) | 69.5 | 70.3 | 71.0 | 73.4 | 75.8 | 74.3 | 78.4 | 84.5 | 92.4 |
| Patient 2(5.63 BU/mL) | 60.7 | 60.3 | 61.0 | 61.6 | 62.9 | 67.6 | 70.3 | 74.1 | 77.6 |
| Patient 2(2.81 BU/mL) | 50.5 | 50.8 | 51.1 | 51.1 | 52.0 | 57.2 | 59.0 | 60.5 | 63.3 |
| Patient 3(1.6 BU/mL) | 52.0 | 52.8 | 52.3 | 51.9 | 51.7 | 53.1 | 54.4 | 55.7 | 58.9 |
| Patient 4(1.6 BU/mL) | 45.9 | 46.1 | 46.2 | 46.2 | 47.2 | 47.5 | 47.9 | 48.6 | 48.9 |
| Patient 4(8.8 BU/mL) | 88.8 | 91.8 | 91.1 | 92.8 | 92.9 | 95.4 | 96.2 | 96.8 | 99.5 |
| Patient 4(17.6 BU/mL) | 110 | 111 | 112 | 113 | 117 | 116 | 118 | 121 | 126 |
| Patient 4(4.4 BU/mL) | 52.1 | 53.5 | 54.3 | 53.9 | 54.2 | 51.7 | 55.2 | 56.0 | 56.7 |
|  | FV: C (%) | | | | | | | | |
| Patient1(1.6 BU/mL) | 45 | 42 | 43 | 40 | 34 | 34 | 25 | 18 | 11 |
| Patient 1(8.8 BU/mL) | 36 | 27 | 23 | 19 | 11 | 14 | 8 | 5 | 2 |
| Patient 1(17.6 BU/mL) | 34 | 22 | 16 | 11 | 8 | 7 | 3 | 2 | 3 |
| Patient 2(1.6 BU/mL) | 25 | 25 | 25 | 26 | 25 | 25 | 25 | 24 | 23 |
| Patient 2(8.8 BU/mL) | 12 | 12 | 11 | 10 | 7 | 7 | 6 | 5 | 5 |
| Patient 2(17.6 BU/mL) | 6 | 6 | 6 | 6 | 5 | 5 | 5 | 4 | 3 |
| Patient 2(22.5 BU/mL) | 6 | 6 | 5 | 5 | 4 | 5 | 4 | 4 | 3 |
| Patient 2(11.3 BU/mL) | 11 | 11 | 10 | 10 | 6 | 6 | 5 | 5 | 4 |
| Patient 2(5.63 BU/mL) | 18 | 17 | 16 | 15 | 14 | 13 | 11 | 7 | 6 |
| Patient 2(2.81 BU/mL) | 25 | 23 | 23 | 21 | 21 | 17 | 16 | 14 | 14 |
| Patient 3(1.6 BU/mL) | 27 | 27 | 26 | 27 | 25 | 24 | 25 | 24 | 21 |
| Patient 4(1.6 BU/mL) | 24 | 24 | 24 | 23 | 23 | 23 | 22 | 22 | 20 |
| Patient 4(8.8 BU/mL) | 11 | 10 | 10 | 10 | 7 | 7 | 7 | 7 | 6 |
| Patient 4(17.6 BU/mL) | 4 | 4 | 4 | 4 | 4 | 4 | 4 | 4 | 4 |
| Patient 4(4.4 BU/mL) | 18 | 16 | 16 | 15 | 16 | 17 | 14 | 14 | 14 |
|  | PT mixing time (s) | | | | | | | | |
| Patient 1(1.6BU/mL) | 13.9 | 14.1 | 14.2 | 14.2 | 15.3 | 15.8 | 16.3 | 17.9 | 22.8 |
| Patient 1(8.8 BU/mL) | 16.5 | 17.9 | 18.6 | 20.5 | 23.8 | 22.3 | 24.9 | 27.1 | 30.0 |
| Patient 1(17.6 BU/mL) | 17.8 | 19.8 | 22.3 | 23.6 | 24.8 | 26.1 | 28.9 | 30.1 | 30.3 |
| Patient 2(1.6BU/mL) | 21.9 | 21.9 | 21.8 | 22.0 | 22.3 | 22.0 | 22.3 | 22.6 | 22.9 |
| Patient 2(8.8 BU/mL) | 27.1 | 27.4 | 27.6 | 28.0 | 28.5 | 27.6 | 28.4 | 29.6 | 31.3 |
| Patient 2(17.6 BU/mL) | 27.9 | 28.9 | 29.2 | 30.4 | 31.3 | 29.4 | 30.3 | 31.9 | 34.9 |
| Patient 2(22.5 BU/mL) | 27.7 | 28.6 | 28.7 | 29.5 | 32.1 | 29.8 | 31.6 | 32.7 | 35.1 |
| Patient 2 (11.3 BU/mL) | 25.8 | 26.0 | 26.2 | 26.7 | 28.3 | 28.6 | 29.8 | 30.9 | 32.3 |
| Patient 2 (5.63 BU/mL) | 23.6 | 23.8 | 23.9 | 24.3 | 24.5 | 25.1 | 26.2 | 26.9 | 27.5 |
| Patient 2(2.81 BU/mL) | 22.6 | 22.9 | 23.1 | 23.2 | 23.4 | 25.5 | 26.0 | 26.3 | 26.8 |
| Patient 3(1.6 BU/mL) | 22.6 | 22.7 | 22.7 | 22.9 | 23.0 | 23.4 | 23.8 | 24.5 | 25.6 |
| Patient 4(1.6 BU/mL) | 21.3 | 21.7 | 21.8 | 21.7 | 22.0 | 22.8 | 23.2 | 23.6 | 24.5 |
| Patient 4(8.8 BU/mL) | 28.8 | 29.4 | 29.3 | 29.4 | 30.0 | 30.9 | 31.6 | 32.4 | 33.5 |
| Patient 4(17.6 BU/mL) | 32.1 | 32.3 | 32.5 | 33.3 | 34.1 | 34.6 | 34.7 | 35.0 | 35.3 |
| Patient 4(4.4 BU/mL) | 26.7 | 27.3 | 27.5 | 27.5 | 28.0 | 27.9 | 28.2 | 28.5 | 30.3 |

**Supplementary Table S1** PT‑mixing time, APTT‑mixing time and residual FV activity of 15 diluted plasma samples derived from four patients with acquired factor V inhibitors under room‑temperature (RT) and 37 °C incubation at different time points.

Notes:

Except for Patient 3, undiluted plasma APTT mixing values of the other three patients exceeded 180 s at room temperature. Similarly, residual FV activity of these undiluted samples fell below the detection limit and was therefore excluded from the table.

Patient 1 had a baseline FV inhibitor titer of 17.6 BU/mL, which was serially diluted to 8.8 BU/mL and 1.6 BU/mL.

Patient 2 had a baseline titer of 90 BU/mL, serially diluted to 22.5, 17.6, 11.3, 8.8, 5.63, 2.81, and 1.6 BU/mL.

Patient 3 had an undiluted titer of 1.6 BU/mL.

Patient 4 had a baseline titer of 274 BU/mL and was serially diluted to 17.6, 8.8, 4.4, and 1.6 BU/mL.

**Supplementary Table S2** Spontaneous thermal degradation data of inhibitor‑free pooled control plasma incubated at room temperature and 37 °C for 0–120 min.

| control samples | Incubation RT (min) | | | | | Incubation 37℃(min) | | | |
| --- | --- | --- | --- | --- | --- | --- | --- | --- | --- |
|  | 0 | 15 | 30 | 60 | 120 | 15 | 30 | 60 | 120 |
| APTT time (s) | 43.3 | 43.6 | 43 | 43.1 | 43.3 | 43.2 | 43.6 | 43 | 42.8 |
| FV activity (FV: C, %) | 16 | 16 | 16 | 16 | 17 | 16 | 16 | 15 | 16 |
| PT time(s) | 20 | 20.1 | 20.1 | 20.1 | 20 | 20 | 20.3 | 20.2 | 20.1 |

Notes:

Pooled inhibitor‑free normal pooled plasma (NPP) was collected from 20 healthy donors with normal coagulation profiles. To reproduce identical dilution gradients as patient plasma for matrix matching, NPP was serially diluted with STA‑Deficient V reagent (FV‑deficient plasma). All diluted NPP aliquots were incubated in parallel with patient samples under consistent time‑temperature conditions. Imidazole buffer was used exclusively in the Bethesda assay for inhibitor quantification. This parallel control setup quantified spontaneous thermal degradation of native FV protein and helped exclude its confounding influence on FV‑inhibitor‑mediated neutralization assessment.
